# Utilization of Hematological Markers to Distinguish Bile Reflux Gastritis From Helicobacter pylori-Associated Gastritis in Pediatric Patients

**DOI:** 10.7759/cureus.97817

**Published:** 2025-11-25

**Authors:** Shehanshah Muhammad Arqam, Maliha Mahmood, Labiqa Khowaja, Naveed Ul Haq, Farhan Ullah, Akshy Kumar, Muhammad Tayyab Rehman, Raheel Ahmad Sohail, Muhammad Hamza Khan, Ali Khan

**Affiliations:** 1 Medicine, Dow University of Health Sciences, Karachi, PAK; 2 Medicine, Balouchistan Institute of Child Health Services, Quetta, PAK; 3 Medicine, Liaquat University of Medical and Health Sciences, Jamshoro, PAK; 4 Medicine, Khyber Teaching Hospital, Peshawar, PAK; 5 Medicine, Pakistan Navy Ship (PNS) Shifa Hospital, Karachi, PAK; 6 Medicine, Shaikh Zayed Hospital, Lahore, PAK; 7 Medicine, Dow University of Health Sciences, Dow International Medical College, Karachi, PAK; 8 Medicine, Jinnah Postgraduate Medical Centre, Karachi, PAK

**Keywords:** bile reflux gastritis, helicobacter pylori, hematological indices, neutrophil-to-lymphocyte ratio, pediatric gastritis

## Abstract

Gastritis is a frequent pediatric disease and is associated with significant morbidity, nutritional compromise and reduction in the quality of life. Among the etiologies, Helicobacter pylori (H. pylori) gastritis and bile reflux gastritis (BRG) are most frequent though clinically indistinguishable without invasive examinations like biopsy. This prospective observational cross-sectional study was carried out at the Department of Pediatric Gastroenterology, Jinnah Postgraduate Medical Centre (JPMC), Karachi, aiming to elaborate the diagnostic utility of these hematological markers in differentiating BRG from H. pylori gastritis. One hundred twenty children aged five to 15 years with endoscopically and histologically confirmed gastritis were recruited from January to December 2024, of which the BRG (n = 60) and H. pylori gastritis (n = 60) groups had equal number of participants. Research data was gathered with the help of a structured proforma, endoscopic examination and confirmed by biopsy from pretreatment blood samples. The determined hematologic parameters were as follows: hemoglobin (Hb), total leukocyte count (TLC), neutrophil-to-lymphocyte ratio (NLR), platelet-to-lymphocyte ratio (PLR), mean corpuscular volume (MCV), red cell distribution width (RDW) and platelet counts. t-tests, Chi-square tests, Mann-Whitney U tests and receiver operating characteristic (ROC) curve analyses were used for statistical analysis.

The children with H. pylori gastritis demonstrated lower levels of hemoglobin (10.9±1.2 g/dL vs 11.8±1.3 g/dL, p=0.001), MCV (80.1±5.7 fL vs 82.6±6.2 fL, p=0·04) and higher RDW (15.6±2.0% vs 14.5±1.9%, p=0.003), indicating iron-deficiency with microcytic anemia in the former group than the latter groups respectively with no such differences seen among patients who were Helicobacter free. On the other hand, NLR was significantly higher in BRG patients (2.6±0.8 vs 2.1±0.7, p=0.001), reflecting an increased neutrophil-mediated inflammation. PLR was higher in H. pylori gastritis (148.7±34.2 vs 131.5±29.1, p=0.02), whereas platelet counts were similar between the two groups (p=0.21). By ROC analysis, NLR (area under the curve (AUC)=0.72) and RDW (AUC=0.70) were the best discriminative factors, as well as hemoglobin level (AUC=0.68).

These results indicated that the simple and inexpensive hematological parameters NLR, RDW, and Hb could have clinical application value as adjuncts for distinguishing between BRG children and H. pylori gastritis patients. An approach like this might reduce the use of invasive procedures, increase diagnostic accuracy, and help in clinical decision making, particularly in low-resource health care settings.

## Introduction

Gastritis in children is a frequent and clinically significant disease that causes substantial morbidity, nutritional compromise, and decreased quality of life. Children with recurrent abdominal pain (RAP), vomiting, bloating, nausea, and poor appetite experience chronic gastritis when subjected to diagnostic evaluation. Of all possible causes, Helicobacter pylori gastritis and bile reflux gastritis are the two most frequently surface etiologies in pediatric population [1]. The problem for physicians is not only in the identification of gastritis but in the validation of the diagnosis, since the therapy for infectious and chemical etiologies is fundamentally different. H. pylori infection is one of the most common chronic bacterial illnesses in the world. It has been shown to be associated with chronic active gastritis, peptic ulcer disease, and, later in life, gastric carcinoma [2]. The microbe is usually acquired in kids and remains asymptomatic unless it is eradicated with a combination of antibiotics and proton pump inhibitors. In children, active Helicobacter pylori gastritis is related with chronic abdominal pain, anorexia, iron deficiency anemia, and growth failure. Histologically information, it generates a characteristic lymphocytic and neutrophilic infiltration of the gastric mucosa with the development of lymphoid aggregates and follicles. H. pylori can cause systemic immune system activation, in the past, modifications in peripheral hematological markers and children have been published [3].

Bile reflux gastritis (BRG), meanwhile, is triggered by the retrograde movement of bile and duodenal content into the stomach. Unlike an infectious agent, this duodenum gastric reflux chemically damages the gastric epithelium. BRG has been documented in children with a history of gastric surgery as well as in non-operated stomachs where disturbances such as pyloric dysfunction or motility may be at play [4]. The clinical spectrum of BRG symptoms is similar to that of H. pylori gastritis, including upper abdominal pain, vomiting, nausea, and weight loss. There is, nevertheless, a characteristic discrepancy in the histological picture: BRG displays extensive foveolar hyperplasia and surface damage, often coupled with epithelial regeneration, but the dense lymphoid aggregates in H. pylori are consistently absent [5]. Since even the endoscopic picture of the two conditions may convey asymptomatic and comparable appearances, the deciding factor must be a confirmatory biopsy. Despite this, although endoscopic biopsy is regarded as the gold standard, the procedure is associated with certain limitations. It is an invasive, expensive test that requires anesthesia in the majority of pediatric patients. Microscopy availability is limited in most low-resource settings, and many parents are reluctant to have their children undergo this form of test, given its significant invasiveness. Therefore, simple, low-cost, non-invasive diagnostic aids for distinguishing BRG from H. pylori are essential [6]. For example, one of the most promising may be hematological indices based on a simple complete blood count. CBC is a low-cost, low-risk test that is widely accessible. Indices such as the neutrophil-to-lymphocyte ratio (NLR), platelet-to-lymphocyte ratio (PLR), mean platelet volume (MPV), and red cell distribution width (RDW) have been used to identify systemic inflammation in various diseases, ranging from infectious and autoimmune to neoplastic conditions [7]. Since H. pylori causes chronic inflammation and BRG is primarily a chemical injury, assuming these two entities exude distinctive systemic hematological pictures seems quite reasonable.

Although theoretically plausible, the evidence from pediatric cohorts on this matter remains scarce and inconsistent in the available medical literature. Most authors have reported statistically significant elevations of NLR and PLR in children with H. pylori gastritis as compared to a non-infected control group, whereas another study has failed to identify any substantial differences between these indicators in patients with H. pylori and BRG [8]. The third potential indicator, MPV, which reflects platelet activation with overall activation of systemic inflammatory status, has also been researched and demonstrated inconsistent findings. All of the abovementioned inconsistencies might be a result of small-sample studies, study designs with poor homogeneity or insufficient control for confounders, including recently acquired infections or drug interference. This work aims to therefore create high-quality primary data by directly comparing BRG patients against children with biopsy-proven H. pylori gastritis and establish the role of routine blood testing in complementing these differential diagnoses [9]. The study’s main goal is thus to comparatively analyze a plethora of hematological parameters represented by white blood cell counts, neutrophil counts, lymphocyte counts, hemoglobin (Hb), platelet counts, NLR, PLR, and MPV in children with BRG and H. pylori gastritis using endoscopy and histology as the reference or gold standard [10]. Other objectives will be to establish the sensitivity, specificity, and predictive accuracy of such hematological indices in facilitating the differentiation between these clinical variants.

In children with H. pylori gastritis, higher inflammation indicators, especially NLR and PLR, than in their counterparts with BRG are assumed to be found while MPV changes would be less evident [11]. The study described here offers a solution to the common diagnostic problem in pediatric gastroenterology. Through the investigation of accessible and inexpensive hematological parameters as diagnostic adjuncts, this study may help to diminish dependency on invasive procedures, assist in clinical decision-making and tailor treatment modalities with children suffering from gastritis. In low-resource pediatric health care settings, this type of approach may enhance access to care, reduce expenses for both the system and patients, and mitigate risks inherent to pediatric endoscopy.

## Materials and methods

This study employed a prospective observational cross-sectional design to evaluate hematological parameters in differentiating BRG from Helicobacter pylori gastritis among children. The methodology outlines the study setting, sampling, inclusion and exclusion criteria, data collection procedures, hematological investigations, and statistical analysis applied.

Study design

This was a prospective observational cross-sectional study which attempted to differentiate BRG from H. pylori-induced gastritis in children based on haematological parameters. The aim of the present study is to offer an original contribution regarding the diagnostic accuracy of inexpensive blood-based simple biomarkers, which are capable of reducing the dependence solely on invasive endoscopic tools.

Study setting

The study was conducted in the Department of Pediatric Gastroenterology, Tertiary Care Hospital, Karachi, which is a tertiary care referral hospital and one of the largest hospitals in Pakistan. The centre is a referral facility for children from all over Sindh and contiguous provinces of Pakistan, thus providing an opportunity for evaluation of a variety of pediatric populations with diverse GI problems.

Study population

The histopathology-confirmed suspected gastritis was defined as children aged five to 15 years, with persistent upper gastrointestinal symptoms (RAP, nausea/vomiting/ dyspepsia) who attended the outpatient as well as inpatient services of the Pediatrics Gastroenterology Department. Recruitment of patients began in January 2024 and was due to be completed in December 2024.

Sample size

Convenience sampling technique was used with a sample size of 120 children. The sample size was calculated according to the previous studies which examined hematological parameters of gastritis, for moderate effect size and a power level of 80% at 95% confidence interval. Among the total sample, 60 children with BRG and 60 children with H. pylori gastritis were diagnosed according to endoscopically and histological observations.

Inclusion criteria

Patients were included in the study if they were five to 15 years old, had at least a four-week history of symptoms compatible with gastritis. Patients with endoscopic evidence of either bile reflux gastritis or Helicobacter pylori-associated gastritis, established by biopsy, were included.

Exclusion criteria

A history of chronic liver disease, celiac disease, inflammatory bowel disease and haematological disorders such as thalassemia or anemia of chronic diseases were excluded from the study. Subjects who had taken antibiotics, proton pump inhibitors, or bismuth compounds during the four weeks prior to endoscopy were excluded. Also, the study excluded patients with a history of previous gastric surgery or an unwillingness or refusal to give consent/assent.

Data collection procedure

A structured proforma was employed to obtain information regarding age, sex, socioeconomic status and clinical history and presenting complaints after obtaining informed consent from the parents/guardians. All patients received upper gastrointestinal endoscopy, in which gastric mucosa was inspected by features of bile reflux (bile staining, erythema and mucosal edema) and biopsies were collected. Histologic verification of H. pylori infection was done with rapid urease test (RUT) and haematoxylin-eosin staining. Blood was drawn before treatment start. Department of Pathology, Jinnah Postgraduate Medical Centre (JPMC) conducted haematological investigations with an automated haematology analyzer.

Hematological parameters measured

A variety of hematological parameters applicable for an assessment of systemic response in gastritis were evaluated. The variables were Hb concentration, expressed in g/dl, total leukocyte count (TLC), expressed in ×10⁹/L and NLR, an indicator of inflammatory activity. This is further relevant to risk prediction as we can evaluate immune and inflammatory status using platelets counts (×10⁹/L) and the PLR.

In addition to these, red blood cell (RBC) indices were evaluated along with mean corpuscular volume (MCV), which indicates the average size of RBCs, and red cell distribution width (RDW), which provides an index of the variability in shape. Together, these markers provide a full framework of hematological profile for a better interpretation involved pathophysiological changes in gastritis subjects.

Statistical analysis

Analysis was conducted using SPSS version 26.0 (IBM Corp., Armonk, NY, USA). Continuous variables including hemoglobin, NLR and PLR were expressed as mean ± standard deviation (SD), whereas categorical variables like gender were represented in frequencies and percentages.

Specifically, statistical methods for comparison between BRG and H. pylori groups were utilized to guarantee the accuracy and reliability of the results. For the statistical analysis of normally distributed continuous variables, we used independent t-test to compare mean values between the two groups. For not normally distributed data, differences of central tendency were evaluated using the Mann-Whitney U test. Categorical variables were analyzed using the Chi-square test to compare proportions and frequencies between groups, after ensuring that the expected frequencies in all cells met the assumptions required for this test.

Apart from these comparison studies, we also performed receiver operating characteristic (ROC) curve analysis to elucidate the diagnostic sensitivity and specificity of selected hematological parameters. 

Statistical significance level was taken as p<0.05 for all analyses. This threshold criteria guaranteed high confidence in the results, thus minimizing the possibility that differences were observed by chance. Application of these statistical techniques helped greatly in interpreting the results of the study.

## Results

The results present demographic characteristics, clinical symptoms, and hematological differences between children with BRG and H. pylori gastritis. Key findings are highlighted through statistical comparisons and diagnostic accuracy analyses to identify significant markers for clinical differentiation.

Demographic details of the study population in Table 1 describe the same. The purpose of the study was to find out the characteristics between children with BRG and H. pylori gastritis based on their baseline presentation features. A total of 120 children were divided into two groups (n = 60). Demographic characteristics including age, sex ratio, BMI, and allele of family history of GI disease were analyzed between groups to understand possible differences in baseline clinical aspects. These features are of particular relevance, because demographic patterns often correlate with susceptibility, course of disease and clinical outcomes in pediatric gastritis.

**Table 1:** Baseline demographic characteristics of children with bile reflux gastritis (BRG) and H. pylori gastritis

Variable	BRG (n = 60)	H. pylori (n = 60)	p-value
Age (years), mean ± SD	11.2 ± 2.8	9.8 ± 3.1	0.02* (t = 2.45)
Male, n (%)	32 (53.3%)	38 (63.3%)	0.28 (χ² = 1.18)
Female, n (%)	28 (46.7%)	22 (36.7%)	0.28 (χ² = 1.18)
BMI (kg/m²), mean ± SD	18.6 ± 2.4	18.1 ± 2.7	0.39 (t = 0.86)
Family history of GI disease, n (%)	12 (20.0%)	22 (36.7%)	0.04* (χ² = 4.18)

The average age of children with BRG at 11.2 ± 2.8 years was significantly greater (p = 0.02) than that of those presenting H. pylori gastritis (9.8 ± 3.1 years), as reported in Table 1. This suggests that BRG occurred after older childhood, while H. pylori infection occurred earlier. Table 1 shows that, in both groups, there was a higher proportion of males: 53.3% BRG and 63.3% H. pylori, but the difference was not statistically significant (p = 0.28). Likewise, the mean ± SD BMI values were similar between groups: 18.6 ± 2.4 (BRG) vs 18.1 ± 2.7 (H. pylori) kg/m² (p = 0.39), which suggested that body composition did not differ among conditions. Family history of gastrointestinal disease tended to be found significantly more often in children with H. pylori-induced gastritis (36.7%) as compared with BRG (20.0%) (p = 0.04), suggesting a possible genetic and environmental predisposition for H. pylori-related gastritis. The distributions of gender and BMI yielded similar values, but the tendency towards male predominance in both patient groups is obvious. These demographic data establish an important background upon which to interpret the subsequent clinical and hematological findings, as they document differences between the patient cohort that has precipitated the observed divergent disease patterns in BRG and H. pylori gastritis.

The clinical presentation of children's gastritis is an important part in discerning BRG from H pylori-related gastritis. The spectrum of symptoms serves not only to mirror the underlying pathological diversities but also for physicians to decide on a diagnosis. Table 2 lists some of the clinical profiles noted in the study population. Both samples had RAP as the single most prominent symptom, whilst differences were noted for vomiting and dyspeptic symptoms, which seemed to be associated with certain etiologies. This also emphasized the chronicity of symptoms and the occurrence of weight loss as indicators of disease severity though the latter were not statistically significant in some comparisons.

**Table 2:** Clinical symptoms in children diagnosed with bile reflux gastritis (BRG) and H. pylori gastritis

Symptom	BRG (n = 60)	H. pylori (n = 60)	p-value
Recurrent abdominal pain, n (%)	49 (81.7%)	53 (88.3%)	0.29 (χ² = 1.12)
Vomiting, n (%)	38 (63.3%)	24 (40.0%)	0.01* (χ² = 6.42)
Dyspepsia, n (%)	31 (51.7%)	42 (70.0%)	0.04* (χ² = 4.22)
Symptom duration >6 months, n (%)	26 (43.3%)	24 (40.0%)	0.72 (χ² = 0.13)
Weight loss >5%, n (%)	9 (15.0%)	17 (28.3%)	0.09 (χ² = 2.86)

The findings demonstrate that RAP was commonly present in both BRG (81.7%) and H. pylori gastritis (88.3%), with no significant difference (p = 0.29). This suggests that despite being nonspecific, abdominal pain is the predominant clinical presentation of childhood gastritis and of little value to differentiate between both groups. Vomiting, however, was much more common in children with BRG (63.3%) than those having H. pylori gastritis (40.0%), p = 0.01, supporting the utility of this symptom as a possible diagnostic aid to differentiate bile-related disease from other causes of pathological vomiting. Dyspepsia presented a reverse trend, having been much higher in the H. pylori group (70.0%) than the BRG (51.7%) with a p-value of 0.04. These new findings stress the need for meticulous clinical profiling as these symptom patterns can offer clues to the specificities of underlying gastritis type even without laboratory or endoscopic stratification.

The two groups did not differ significantly in the chronicity and systemic involvement (p = 0.72), except for a duration of symptoms above six months that was found in sentries, i.e., BRG patients and those with H. pylori reported respectively by 43.3% and 40.0%. This would indicate that chronic gastritis, whatever its cause, is usually manifested by a prolonged clinical course. There was comparable weight loss of over 5% from baseline body weight in 15.0% and in 28.3% of BRG group patients and H. pylori group patients, respectively, but it did not reach statistical significance (p = 0.09). These, along with our findings, highlight the common and unique clinical profiles of gastritis in children and help guide symptom-based differentiation in practice.

The analysis of hematological indices in children with BRG and HPG is considered to be important for disclosing traits of the pathogenesis process. In Table 3, mean ± SD of hematological indices (hemoglobin, TLC, NLR, PLR, MCV, RDW coefficient of variance in percentage) and platelet counts are given. The hematology characteristics between the two groups show the discrepancy between these hematological markers. The parameters were examined in a statistical manner to recognize differences that might be valuable for diagnosis between BRG and H. pylori gastritis.

**Table 3:** Hematological parameters among bile reflux gastritis (BRG) and H. pylori gastritis patients TLC: total leukocyte count, NLR: neutrophil-to-lymphocyte ratio, PLR: platelet-to-lymphocyte ratio, MCV: mean corpuscular volume, RDW: red cell distribution width

Parameter	BRG (Mean ± SD)	H. pylori (Mean ± SD)	p-value
Hemoglobin (g/dL)	11.8 ± 1.3	10.9 ± 1.2	0.001* (t = 3.42)
TLC (×10⁹/L)	8.2 ± 2.1	8.5 ± 2.4	0.460 (t = 0.74)
NLR	2.6 ± 0.8	2.1 ± 0.7	0.001* (t = 3.38)
PLR	131.5 ± 29.1	148.7 ± 34.2	0.020* (t = 2.36)
MCV (fL)	82.6 ± 6.2	80.1 ± 5.7	0.040* (t = 2.09)
RDW (%)	14.5 ± 1.9	15.6 ± 2.0	0.003* (t = 3.05)
Platelets (×10⁹/L)	302 ± 74	318 ± 82	0.210 (t = 1.26)

Decreased hemoglobin levels were significantly more frequent in children with H. pylori gastritis compared to BRG patients (10.9 ± 1.2 vs 11.8 ± 1.3 g/dL, p = 0.001). This result may reflect the fact that subjects infected with H. pylori are at higher risk of anemia, compatible with previous results indicating a relationship between chronic infection by H. pylori and diminished iron absorption, and increased incidence of iron deficiency anemia. In contrast, the two groups did not significantly differ in mean TLC values (p = 0.46), indicating that leukocyte counts may not be a discriminating factor for distinguishing these categories of gastritis. The NLR was higher in BRG patients (2.6 ± 0.8) than in H. pylori gastritis (2.1 ± 0.7, p = 0.001), suggesting that BRG may relate to stronger systemic inflammation with neutrophil provocation exceeding above lymphocytes.

Other hemogram parameters also had a significant difference. Statistically significant differences were observed when comparing children with H. pylori gastritis vs. BRG regarding the PLR level (148.7 ± 34.2 vs. 131.5 ± 29, p = 0,02), thus underlying an enhanced platelet-mediated inflammatory contribution during H. pylori infection. The MCV was also lower in the H. pylori group (80.1 ± 5.7 fL vs 82.6 ± 6.2 fL, p = 0.04), indicating that H. pylori was associated with microcytic anemia. RDW was also higher in the H. pylori group (15.6 ± 2.0% vs. 14.5 ± 1.9%, p = 0,003), pointing to greater heterogeneity of red blood cell sizes which is often seen in nutritional deficiencies and chronic inflammation. Platelet levels, slightly elevated in coexistence with H. pylori gastritis (318 ± 82 ×10⁹/L) compared with BRG (302 ± 74 ×10⁹/L), were not significantly different (p = 0.21). Taken together, the results of Table 3 highlight unique hematological changes in BRG as compared to H. pylori gastritis, and that hemoglobin, NLR, PLR, MCV and RDW are the most differentiating parameters among them.

The aim of this study was to investigate gender-related differences in hematologic parameters in children suffering from BRG and Helicobacter pylori gastritis. It is clinically important to recognize these gender-dependent discrepancies, since sex-related physiological and hormonal aspects can modulate blood indices, inflammatory pattern, and severity of disease. The analysis also gives us some clues about whether hematological markers are the same in males and females and if there are sex-related differences in values for gastritis-related diseases including the standard platelet counts. In Table 4 of male and female patients, the differences were observed in hemoglobin, NLR, RDW, PLR and platelets.

**Table 4:** Gender-stratified analysis of hematological parameters in bile reflux gastritis (BRG) and H. pylori gastritis NLR: neutrophil-to-lymphocyte ratio, PLR: platelet-to-lymphocyte ratio, RDW: red cell distribution width

Parameter	BRG Male (n=32)	BRG Female (n=28)	H. pylori Male (n=38)	H. pylori Female (n=22)	p-value (overall)
Hemoglobin (g/dL)	11.9 ± 1.2	11.7 ± 1.4	10.7 ± 1.3	11.1 ± 1.1	0.002* (F = 4.97)
NLR	2.5 ± 0.7	2.7 ± 0.9	2.0 ± 0.6	2.2 ± 0.7	0.010* (F = 3.64)
RDW (%)	14.4 ± 1.8	14.6 ± 2.0	15.8 ± 2.2	15.3 ± 1.9	0.004* (F = 4.62)
PLR	129.5 ± 28.0	133.7 ± 30.2	146.1 ± 33.1	152.5 ± 35.7	0.030* (F = 3.11)
Platelets (×10⁹/L)	298 ± 70	306 ± 78	314 ± 85	323 ± 79	0.180 (F = 1.58)

The gender-based hematological parameters are shown in Table 4. Mean hemoglobin was significantly higher in BRG patients compared to H. pylori gastritis, with the highest mean level found among BRG males (11.9 g/dL) and the lowest in H. pylori male cases (10.7g/dL). This result may be indicative of a possible effect of the infection by H. pylori in hematopoietic function, especially in men, and is consistent with studies that have found iron deficiency anemia as a common complication from this infection. Conversely, NLR values were increased in both BRG groups versus H. pylori, which indicated more systemic inflammation in BGR cases. Females with BGR presented slightly higher NLR (2.7) than males (2.5), however among H. pylori patients, lower NLR was found in males as compared to female individuals. That expressed in RDW levels where H. pylori male patients had significantly higher RDW (15.8%) than the BRG group (14.4-14.6%). This result is in correlation to the effect of H. pylori on iron metabolism homeostasis and erythropoiesis.

PLR values showed significant gender-related differences, with higher ratios recorded in females for both BRG (133.7) and H. pylori (152.5) groups compared to male subjects. This may imply that females experience a stronger platelet-mediated inflammatory response in gastritis. However, platelet count per se exhibited no statistically significant difference between groups but was marginally higher in females than in males in both sample sets. The global p-value of 0.18 for platelets suggests that the discriminatory power of platelets may not be stable and reliable when assessed on subpopulations by gender or diseases in pediatric gastritis. In summary, sex has an impact on several hematological indices especially hemoglobin level, NLR, RDW and PLR, while platelet count is relatively constant.

The association between hematological parameters and clinical severity of gastritis in our pediatric study group is described here. Symptom severity and abdominal pain frequency were evaluated in association with hemoglobin, NLR, PLR, RDW, and a number of platelets present. These correlations can help to understand the degree of hematologic markers with severity of clinical presentations and is useful for identifying disease severity in children with gastritis. These results are presented in Table 5, and also clearly indicate both significant and non-significant observations.

**Table 5:** Correlation between symptom severity and hematological parameters NLR: neutrophil-to-lymphocyte ratio, PLR: platelet-to-lymphocyte ratio, RDW: red cell distribution width

Clinical Severity Index	Parameter	r- Value	p-Value	t-Value
Abdominal pain frequency	Hemoglobin	-0.29	0.03	2.20
Abdominal pain frequency	NLR	0.42	<0.01	3.55
Abdominal pain frequency	PLR	0.18	0.09	1.70
Abdominal pain frequency	RDW	0.38	0.01	2.90
Abdominal pain frequency	Platelets	0.11	0.24	1.20

Table 5 shows that hemoglobin was inversely correlated with the frequency of abdominal pain (r = -0.29, p = 0.03), i.e., children with lower hemoglobin levels had more frequent abdominal pain. By comparison, NLR was significantly correlated with severity of pain (r = 0.42, p < 0.01), suggesting that the greater the systemic inflammation (as indicated by higher NLR) the more severe symptoms experienced. RDW was also positively and significantly correlated with it (r = 0.38, p = 0.01), confirming the utility of RDW as a marker of severity in gastritis. In contrast, PLR was only weakly non-significantly correlated with abdominal pain (r = 0.18, p = 0·09) and platelet level on its own exhibited little correlation (r = 0.11, p = 0·24), illustrating the low value of these as a predictive test in this study setting.

These correlations are well depicted in Table 5 where Hb seems to inversely correlate with symptom frequency; both NLR and RDW present an ascending tendency according to the increase of disease clinical severity. The weak association between PLR, platelet count points out their poor diagnostic performance compared to others. In summary, all these observations demonstrate that although the NLR and RDW are most accurate in reflecting clinical severity of disease among the hematological parameters studied with a contributory role of hemoglobin especially for recurrent abdominal pain presented pediatric gastritis.

ROC curve analysis was utilized to evaluate the diagnostic value of hematologic parameters in differentiating BRG from Helicobacter pylori gastritis. This method was used to calculate the discriminatory power of each marker based on the area under the curve (AUC) and find the best cut-off values according to their sensitivity and specificity. The examined parameters were hemoglobin, NLR, PLR, RDW and platelet count.

It is demonstrated by Table 6 that the AUC of hemoglobin was equal to 0.68 (95% CI: 0.59-0.77), detected at cut-off point ≤11.2 g/dL, and showing sensitivity and specificity values equal to 66.7% and 65% respectively (p=0.002). It reveals that lesser hemoglobin was more expected to be related with H. pylori gastritis rather than with BRG. The NLR was the most efficient parameter for a diagnosis, with an AUC=0.72 (95% CI: 0.63-0.81) at >2.3 cut-off value, sensitivity=70.0%, specificity=68.3% (p140 cut-off, only moderate sensitivity (55.0%) and specificity (58.3%), and p-value which was not significant (p=0.08). Platelet count also had an AUC of 0.57 (95% CI: 0.48-0.66), with a cut-off ≥310 ×10⁹/L, and exhibited low diagnostic power both in sensitivity and specificity terms (50.0% and 55.0%, respectively) (p=0.12). These results are corroborated by the ROC analysis where the hemoglobin and NLR curves fall above, and RDW is entirely separated from the reference line, while PLR and platelets lie close to the diagonal which implies lack of clinical value. Our findings reinforce that, although ordinary blood parameters such as platelet numbers may not serve as good diagnostic markers, inflammation-associated indicators like NLR and RDW are better discriminators of BRG vs. H. pylori gastritis.

**Table 6:** Diagnostic accuracy of hematological parameters in differentiating bile reflux gastritis (BRG) from H. pylori gastritis NLR: neutrophil-to-lymphocyte ratio, PLR: platelet-to-lymphocyte ratio, RDW: red cell distribution width, AUC: area under the curve

Parameter	AUC (95% CI)	Cut-off	Sensitivity (%)	Specificity (%)	p-value
Hemoglobin	0.68 (0.59–0.77)	≤11.2 g/dL	66.7	65.0	0.002* (Z = 3.09)
NLR	0.72 (0.63–0.81)	>2.3	70.0	68.3	<0.001* (Z = 3.85)
PLR	0.59 (0.49–0.69)	>140	55.0	58.3	0.080 (Z = 1.74)
RDW	0.70 (0.61–0.79)	≥15%	68.3	65.0	0.001* (Z = 3.33)
Platelets	0.57 (0.48–0.66)	≥310 ×10⁹/L	50.0	55.0	0.120 (Z = 1.55)

## Discussion

In this study, we aimed to distinguish BRG from H. pylori-associated gastritis in children using hematologic indices of Hb, NLR, PLR, RDW, and other RBC parameters. Our results demonstrate a number of clinically important differences that are in concordance with, and some in opposition to, the literature previously published for pediatric and adult age groups.

In our study, patients with BRG were significantly older than those presenting H. pylori gastritis (11.2 versus 9.8 years; p = 0.02). This observation would indicate that BRG may take place later in life, the reason being a more prolonged exposed the mucosa ulcered by gastroesophageal reflux and a different gastric emptying. These results are consistent with [12] in that BRG was more common among children older during endoscopy for upper gastrointestinal symptoms and H. pylori gastritis, which clustered around younger school age groups. Positive family history for gastrointestinal diseases was found more frequently in H. pylori-infected children (36.7%) than those with BRG (20.0%), consistent with [13], indicating that household transmission is still a major risk factor for pediatric H. pylori acquisition. Accordingly, our findings validate the age and familial patterns of H. pylori infection as well as provide new evidence for BRG in relatively older pediatric populations.

There were also dissimilarities in the clinical range of the two groups. Vomiting was more common in BRG, and dyspepsia and early satiety were more prevalent in H. pylori gastritis. Agreeing with this observation, Shi et al., 2022 identified that BRG frequently presents with prolonging postprandial nausea and bilious vomiting compared to H. pylori gastritis which results in epigastric complaints and dyspeptic symptoms. Loss of weight was more frequent among H. pylori cases in our series but was not statistically significant. Assa et al., 2022 reported that chronic H. pylori gastritis may impair nutrient absorption and cause subtle growth impairment in children, which is compatible with our findings in the present study [14]. Collectively, these procedures indicate that symptom complexes could provide preliminary diagnostic leads prior to invasive studies.

The most important discovery in this study was the hematological difference between these two groups. The children with H. pylori gastritis had significantly lower levels of hemoglobin and higher RDW, while those with BRG showed a higher NLR. The decrease in hemoglobin level of H. pylori-infected patients was consistent with the reported strong correlation between the infection and iron deficiency anemia. Hamdan et al., 2022 evidenced this pattern in a large pediatric cohort where H. pylori infection induced anemia caused by reduced iron absorption and longstanding mucosal inflammation [15]. Similarly, Motupalli and Oroszi, 2024 found that increase in RDW levels could be an early marker to detect iron-deficient erythropoiesis in a H. pylori gastritis [16]. Our study confirms these results, suggesting that RDW and hemoglobin could be of special interest as biomarkers of H. pylori-associated gastritis in children.

On the other hand, NLR was higher in patients with BRG compared to non-BRG, indicative of intense neutrophil-induced inflammatory reaction. Although the majority of investigations into NLR have been directed at H. pylori gastritis, recent work from Ergun et al., 2023 demonstrated that duodenum gastric reflux causes significant neutrophil infiltration of the gastric mucosa [17]. Our findings add to this by showing that systemic measures of inflammation, i.e. NLR are likewise reflective of the local immune response. The PLR was higher in H. pylori gastritis in this study which was consistent with the results from Sağlam and Civan, 2023 who found elevated PLR in children with chronic H. pylori infection, which could be associated with low-grade systemic inflammation [3].

Of note, MCV in the H. pylori group was lower, and microcytic anemia was found. This is consistent with the reports of Fekri et al., 2020 who reported low MCV as a common haematological aberration in children with infection [18]. Collectively, these findings are supportive of the idea that hematological changes in response to H. pylori were mediated mainly through iron deficiency, and for BRG more by neutrophil-mediated inflammatory alterations (represented as increased NLR).

These relationships were strengthened by sex differences. Male H. pylori patients had lower hemoglobin and higher RDW, and female BRG patients had more NLR than their respective H. pylori groupings. This gender gap is consistent with the one found in the study by Eyoum Bille and Kouitcheu Mabeku, 2022, who found that H. pylori‐infected boys were at higher risk than those noninfected to develop anemia, which could be explained by different dietary iron intake and growth rate between the sexes [19]. In contrast, a more hale-provoked profile in female BRG patients is less known, but it could reflect immunological disparities between sexes as alluded for pediatric autoimmune diseases.

In this regard, NLR and RDW appeared to be positively correlated with the frequency of abdominal pain, while hemoglobin was negatively correlated. This trend supports the clinical value of these indices. Likewise, Walle et al., 2025 reported that high RDW was associated with the severity of gastrointestinal symptoms in anemic children with H. pylori infection [20]. The absence of a significant relation between the platelet counts and symptom severity in our study is consistent with those of Săsăran et al., 2020, who found that the platelet indices were not as reliable in indicating disease activity of paediatric gastritis [21].

Third, the ROC curve analysis revealed NLR and RDW as the best discriminants for distinguishing between BRG and H. pylori gastritis with AUC of 0.72 and 0.70 respectively. Hemoglobin presented a moderate diagnostic performance, while PLR and platelet count showed less efficiency. These results are consistent with findings reported by Koc and Gedikli, 2022, who demonstrated NLR and RDW to have better ROC performance than platelet indices in differentiating inflammatory from infectious gastritis [22]. Significantly, our findings expand on these observations to a pediatric population and make a distinction between BRG and conventional H. pylori gastritis, which has been discussed less frequently in previous reports.

In conclusion, our results show that hematological parameters are useful as reliable markers to distinguish BRG from H. pylori gastritis in children. Decreased hemoglobin and MCV and an increased RDW support the diagnosis of H. pylori-associated gastritis, whereas higher NLR appears to favor BRG. The present findings are in line with available evidence for pediatrics and adults whereas we also propose novel perspectives on gender-dependent variations and diagnostic considerations.

This study also has limitations that should be taken into account when interpreting the results. The study was carried out at one tertiary care center, so findings may not be generalizable to more diverse pediatric settings. While appropriate for primary comparisons, that sample is relatively small when stratified into categories such as gender or symptom severity, likely reducing power. There are also limitations associated with the cross-sectional study design, which prevent causality inference or following the changing of hematological parameters in relation to treatment or disease progression.

Furthermore, although the main confounders were not included, we could not control for potential unidentified nutritional deficiencies, infections or micronutrient disproportions, which might have affected hematological levels. There was no objective evidence of bile reflux such as Bilitec measurement or impedance used and the supposition of the presence of biliary reflux was based on endoscopic and histological findings. Lastly, the diagnostic cut-off points as determined from ROC analysis were based on the same patient population and need further validation in diverse multi-center cohorts for clinical utility.

## Conclusions

The present research noted the clinical utility of common hematological indicators in the differentiation of BRG and H. pylori gastritis, two diseases sharing similar symptoms but with wholly different etiologies and treatments. Findings highlight the potential of CBC-based indices as convenient, non-invasive, and cost-effective diagnostic tools, particularly given that invasive endoscopy could be unsustainable or restricted in resource-challenged pediatric healthcare settings. Moreover, the association of hematological markers with the severity of symptoms (eg, negative correlation for hemoglobin and abdominal pain and positive for NLR and RDW with severity) indicate their wider applicability in assessing clinical progression. By proving the extent to which straightforward blood work may yield important diagnostic information, this study supplies an evidence-based model capable of saving resources while minimizing the pediatric endoscopy-related risks and maximizing early therapeutic efforts. Larger prospective studies in multicentric settings with long-term follow-ups are needed to confirm these results, fine-tune diagnostic cut-off points, and examine the role of hematological indices in established pediatric gastroenterology protocols.

## Appendices

A structured proforma was employed to obtain information regarding each participant’s demographic and clinical characteristics after obtaining informed consent from parents or guardians (Table 7).

**Table 7:** Data Collection Proforma

Section	Variable	Response Type/ Options
1. Demographic Details	Name / ID No.	Text/Numeric
	Age	Years
	Sex	Male/Female
	Socioeconomic Status	Low/Middle/High
2. Clinical History and Presenting Complaints	Duration of Symptoms	Weeks/Months
	Abdominal Pain	Yes / No
	Frequency of Abdominal Pain	Number per Week
	Vomiting	Yes / No
	Nature of Vomiting	Specify Type
	Frequency of Vomiting	Number per Day
	Dyspepsia	Yes / No
	Nausea	Yes / No
	Weight Loss (>5% Body Weight)	Yes / No
	Family History of Gastrointestinal Disease	Yes / No
3. Examination and Diagnostic Findings	Endoscopic Finding: Bile Staining	Present / Absent
	Endoscopic Finding: Mucosal Erythema	Present / Absent
	Endoscopic Finding: Edema	Present / Absent
	Biopsy Findings (H. pylori)	Positive / Negative
	Final Diagnosis	BRG/H. pylori Gastritis
4. Hematological Parameters	Hemoglobin	g/dL
	Total Leukocyte Count (TLC)	×10⁹/L
	Neutrophil Count	%
	Lymphocyte Count	%
	Neutrophil-to-Lymphocyte Ratio (NLR)	Numeric Value
	Platelet-to-Lymphocyte Ratio (PLR)	Numeric Value
	Mean Corpuscular Volume (MCV)	fL
	Red Cell Distribution Width (RDW)	%
	Platelet Count	×10⁹/L
